# Cross-Modal Audiovisual Modulation of Corticospinal Motor Synergies in Professional Piano Players: A TMS Study during Motor Imagery

**DOI:** 10.1155/2019/1328453

**Published:** 2019-04-04

**Authors:** Simone Rossi, Danilo Spada, Marco Emanuele, Monica Ulivelli, Emiliano Santarnecchi, Luciano Fadiga, Domenico Prattichizzo, Alessandro Rossi, Daniela Perani

**Affiliations:** ^1^Department of Medicine, Surgery and Neuroscience, Unit of Neurology and Clinical Neurophysiology, Siena Brain Investigation and Neuromodulation Lab (Si-BIN Lab), University of Siena, Italy; ^2^Department of Brain and Behavioral Sciences, University of Pavia, Italy; ^3^Section of Human Physiology, University of Ferrara, Italy; ^4^Berenson-Allen Center for Noninvasive Brain Stimulation, Department of Neurology, Division of Cognitive Neurology, Beth Israel Deaconess Medical Center, Harvard Medical School, Boston, MA, USA; ^5^Center for Translational Neurophysiology, Istituto Italiano di Tecnologia, Italy; ^6^Human Centered Robotics Group, SIRSLab, Department of Information Engineering and Mathematics, University of Siena, Italy; ^7^Vita Salute San Raffaele University, Division of Neuroscience, Scientific Institute San Raffaele, Milan, Italy

## Abstract

Transcranial magnetic stimulation was used to investigate corticospinal output changes in 10 professional piano players during motor imagery of triad chords in C major to be “mentally” performed with three fingers of the right hand (thumb, index, and little finger). Five triads were employed in the task; each composed by a stable 3rd interval (C4-E4) and a varying third note that could generate a 5th (G4), a 6th (A4), a 7th (B4), a 9th (D5), or a 10th (E5) interval. The 10th interval chord was thought to be impossible in actual execution for biomechanical reasons, as long as the thumb and the index finger remained fixed on the 3rd interval. Chords could be listened from loudspeakers, read on a staff, or listened and read at the same time while performing the imagery task. The corticospinal output progressively increased along with task demands in terms of mental representation of hand extension. The effects of audio, visual, or audiovisual musical stimuli were generally similar, unless motor imagery of kinetically impossible triads was required. A specific three-effector motor synergy was detected, governing the representation of the progressive mental extension of the hand. Results demonstrate that corticospinal facilitation in professional piano players can be modulated according to the motor plan, even if simply “dispatched” without actual execution. Moreover, specific muscle synergies, usually encoded in the motor cortex, emerge along the cross-modal elaboration of musical stimuli and in motor imagery of musical performances.

## 1. Introduction

Since the musculoskeletal system is highly redundant, the motor system is thought to employ a restricted set of modular commands, or synergies, to accomplish both automatic and goal-directed actions [1]. For example, using principal component analysis (PCA), it has been demonstrated that few principal components account for a great amount of variance in the hand's degrees of freedom (i.e., joint angles) during maintenance of static hand postures [2]. This strategy aims to reduce the dimensionality of motor commands with valuable computational advantages. Several lines of evidence both in humans and nonhuman primates suggest that motor synergies are implemented in the corticospinal outputs. In rhesus macaques, a motor cortical region containing neurons that specify functional synergies of distal and proximal muscles has been identified [3, 4]. In addition, electrical microstimulation of monkey's motor cortex evokes complex and highly coordinated movements across multiple joints, matching common gestures in the monkey's natural repertoire [5]. Moreover, in a kinematic study addressing hand's movements evoked by transcranial magnetic stimulation (TMS), PCA revealed few synergies resembling those extracted from volitional motion of the hand [6]. Recently, in a study combining kinematic, electromyographic and neuroimaging recordings, synergies involved in several hand postures were successfully predicted by neural activation pattern in the motor cortex [7]. These findings suggest that neural assemblies in the motor cortex are connected in a complex way to the periphery and might contribute to arm movements that require the coordinated activation of some muscles and relaxation of others. Therefore, the control of movements in the motor cortex might be organized in terms of behaviorally useful actions.

By TMS of the motor cortex, it is possible to dissect the engagement of the motor system in planned [8] or executed actions [9] in relation to several physiological properties. Therefore, TMS is the most appropriate tool to investigate changes of corticospinal output during a variety of cognitive and motor tasks involving the primary motor cortex and the connected brain regions [10, 11]. Motor plans dispatched, but not executed, towards the “prime mover” of the imagined movement involving either wrist or intrinsic hand muscles can be disclosed by TMS in healthy human subjects [12–14]. Together with neuroimaging investigations, these studies converge on the conclusion that neural networks underpinning imagined and executed actions largely overlap and functionally engage the primary motor cortex as a final effector area, although to a lesser extent for motor imagery than for execution [15–17].

In the musical domain, neuroimaging investigations comparing piano execution with imagery showed overlapping activations in a widespread frontoparietal network [18] including the premotor areas, the precuneus, and the medial part of the left intraparietal sulcus [19, 20], but surprisingly, the involvement of the sensorimotor cortex during imagery is still controversial [19, 21]. Although TMS can disclose the causal involvement of brain regions in cognitive and behavioral tasks, studies investigating motor imagery employing this technique in piano players, and in musicians in general, have been seldom performed [22]. Moreover, the involvement of the sensorimotor cortex during the imagination of musical performance execution would be expected in skilled musicians, given their natural ability in translating audiovisual musical stimuli into motor commands [23].

Here, we asked whether a cross-modal modulation of the corticospinal output occurs in professional piano players during motor imagery of triad chords by manipulating the sensory modality through which chords are prompted (i.e., visual, auditory, or audiovisual). Triad chords were "performed" with three fingers: the thumb (controlled by the Abductor Pollicis Brevis (APB) muscle), the index finger (controlled by the Flexor Digitorum Superficialis (FDS) muscle, beyond the first dorsal interosseous muscle), and the little finger (controlled by the Abductor Digiti Minimi (ADM) muscle and by the synergic wrist extensor muscles Extensor Communis Digitorum (ECD) muscle).

We reasoned that TMS-evoked responses could reflect the recruitment of motor synergies involving the ADM muscle (i.e., the prime mover of the experimental task, therefore the one that should better differentiate the various experimental conditions) and the other muscles included into the mental representation of the progressive hand extension, as required by the progressively larger musical intervals (from the 5^th^ to the extremely demanding for most subjects 10^th^ interval). Moreover, we added a condition in which the actual execution of the chord to be mentally imagined was kinetically impossible for the tested subjects (i.e., a 10^th^ interval chord, while keeping the thumb and the index finger on the 3^rd^ interval keys).

## 2. Subjects and Methods

### 2.1. Participants

The sample was composed of 12 fully right-handed professional piano players (7 males, age range: 22-41 years) with more than 12 years of 4-hour daily piano practice and master degree at the Conservatory “Luigi Cherubini” (Florence, Italy) and at the Istituto Superiore di Studi Musicali “Rinaldo Franci” (Siena, Italy). Due to data corruption, 2 subjects (1 male) were discarded from analysis. The protocol was approved by the Local Ethics Committee, and the subjects gave their written informed consent to participate. None had contraindications to TMS [24].

### 2.2. Paradigm and Stimuli

**Figure 1:**
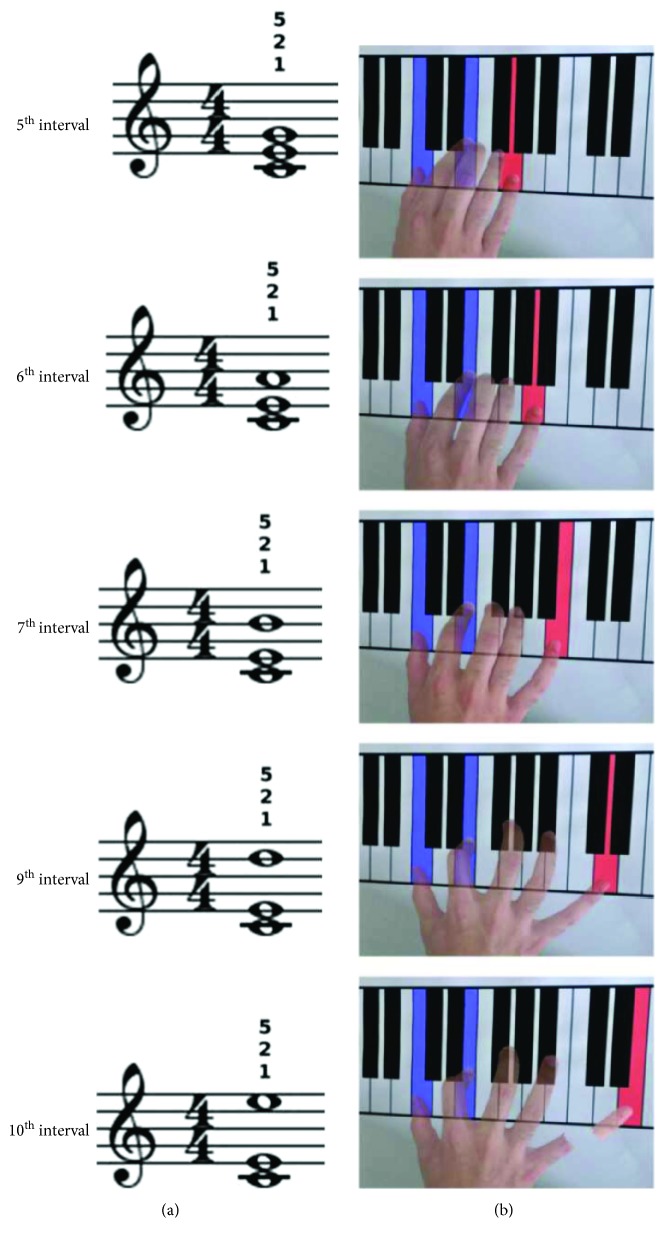
Experimental sketch. Triads were prompted through a visual, auditory, or audiovisual stimulus. The visual stimulus was the chord written on a stave (a); small numbers over each stave denote the required fingering for each chord (i.e., 1 stands for the thumb, 2 for the index finger, and 5 for the little finger). Participants were instructed on the fingering to employ before initiating the experiment; therefore, no further indication on the fingers to be used were administered as chords were prompted. The imagined extension of the hand increased across chords (b); the broken finger in the 10^th^ interval chord denotes that the actual execution was impossible due to biomechanical constraints.

Participants sat in front of a table where a piano keyboard was depicted (Figure 1) with their arms fully relaxed. The task consisted into performing the mental execution of 5 chords in C major with the right hand on the presented keyboard. Chords were formed by a fixed 3^rd^ interval (C4-E4) topped by a varying third note, resulting in a 5^th^ (G4), 6^th^ (A4), 7^th^ (B4), 9^th^ (D5), or 10^th^ (E5) interval (Figure 1, column 1). The actual execution of the 10^th^ interval chord was thought to be kinetically impossible due to biomechanical constraints. An auditory, visual, or bimodal audiovisual stimulus prompted the imagery of 1 of the 5 chords at each trial. In particular, 5 auditory stimuli were used, corresponding to the 5 chords digitally recorded through a MIDI-controlled sampler playing real piano sounds. Short staves with the chord to be imagined written on it served as visual stimuli. In the audiovisual condition, the two stimuli were delivered together at the same time. Stereo loudspeakers and a PC monitor were used for auditory and visual stimuli administration, respectively. Each stimulus lasted 3 s and was preceded by a warning acoustic signal lasting from 1 to 2 s. An experimental constraint was that subjects were always required to mentally execute the triads using the thumb and the index finger for C4 and E4, respectively, and the fifth finger for the third note (Figure 1). All subjects were given the opportunity to train with the task before starting the experiment, until they were able to perform the imagery without showing any electromyographic activity in the recorded muscles. Participants were instructed to initiate motor imagery of the visually, auditory, or audiovisually prompted chords immediately after the appearance of the corresponding stimulus. The appearance of an empty stave served as a control condition during which no motor imagery was required. All conditions and musical intervals were repeated 12 times in a fully randomized order. The duration of the experiment ranged from 45 to 60 minutes.

### 2.3. Neurophysiological Procedures

A circular nonfocal coil connected with a Magstim 200 monophasic stimulator (Magstim, Whiteland, Dyfed, UK) was positioned on the vertex with its handle pointing backwards. The cortical representations of the right Extensor Communis Digitorum (ECD), Flexor Digitorum Superficialis (FDS), Abductor Pollicis Brevis (APB), and Abductor Digiti Minimi (ADM) were targeted within the left motor cortex. Motor-evoked potentials (MEPs) were recorded by means of surface electrodes placed with a tendon-belly montage and connected to a four-channel Neuropack electromyograph (Nihon Kohden, Tokyo, Japan) sampling at 20 kHz with a bandpass 20 Hz-5 kHz filter. The choice of a nonfocal coil guaranteed stable simultaneous responses from all the considered forearm and hand muscles, even if positioned outside the hot spot of each muscle [25]. To the same aim, the intensity of the TMS pulse was adjusted to obtain fairly stable motor-evoked potentials (MEPs) simultaneously from the right ECD, FDS, APB, and ADM muscles. The intensity of TMS was set at 110-120% of the resting motor threshold, defined as the minimal intensity to produce MEPs of less than 100 *μ*V in the target muscles with 50% probability.

In order to minimize habituation, each TMS pulse was delivered following a jittered time interval ranging from 1 s to 2 s from the deployment of the prompting stimulus, therefore well outside a simple reaction time that could have biased the resulting MEPs' amplitude [12, 13, 26, 27]. This time served also to monitor the EMG silence in the target muscles in the time preceding the brain stimulation. The EMG silence preceding the TMS pulse was also monitored using an acoustic feedback provided by the EMG recorder. Trials contaminated by EMG or other artifacts were less than 18%, so that 8-10 MEPs per muscle were available for the statistical analyses in each condition.

### 2.4. Data Analysis

For each muscle, the peak-to-peak amplitudes of the MEPs obtained in each experimental condition were averaged and expressed as a percentage of the average MEP amplitude recorded during the control condition.

Since the assumption of normality was violated as assessed by the Shapiro-Wilk test, nonparametric test statistics were adopted. A paired sample permutation test [28] based on a t-statistic was used to perform pairwise comparisons of MEP amplitude for the factor muscle, condition, and chord; the condition by muscle, chord by muscle, and chord by condition by muscle interactions were also evaluated. At least 5000 permutations were run for each comparison. This is considered an appropriate number of permutations for a significance level of 0.05 [29]. *p* values were adjusted using the false discovery rate (FDR) method [30] in order to control familywise error rate.

Nonnegative matrix factorization (NNMF) [31] was used to extract synergies from muscle activity elicited by TMS during motor imagery. The algorithm was fed with input matrices containing 4 columns (i.e., 1 for each recorded muscle) and 10 rows storing the average MEPs' amplitude in each subject during motor imagery of a given chord or under one particular condition. The procedure was repeated for all chords and conditions. Given the number of synergies to extract, NNMF generates two output matrices whose product approximates the original matrix, i.e., a synergy matrix and a matrix of coefficients. An iterative method starting from initial random values of synergy and coefficients is used to minimize the root-mean-squared residual error between the original matrix and the product of the two output matrices. In our case, the algorithm used the multiplicative update rule [32] and ran 50000 times using different random initial values and selected the solution corresponding to the lowest error in reconstruction. The goodness of factorization was assessed by means of the variance account for (VAF) method [33], i.e., by evaluating the ratio of the sum of squared residuals and the sum of squared residual from mean activation. Pairwise comparisons of the extracted synergies were performed for the factor condition and chord. To this end, the similarity between paired synergies was first assessed by means of the dot product of their matrices of coefficients. Then, the measured dot products were compared to the 99^th^ percentile of the distribution of shuffled dot products computed by random labeling of the matrix of coefficients (*p* < 0.01) [34].

## 3. Results

### 3.1. Pairwise Permutation Tests

**Figure 2:**
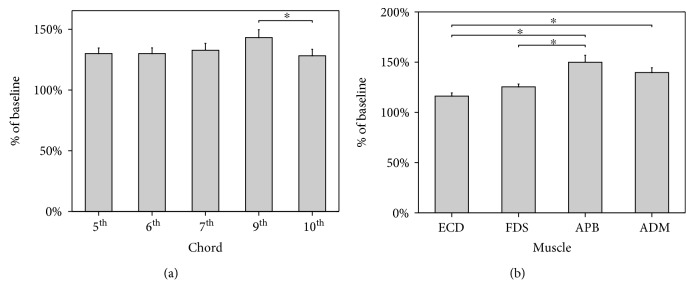
Mean MEPs' peak-to-peak amplitude for each chord (a) and muscle (b) expressed as percent change from baseline. (a) Motor imagery of a 9^th^ interval triad chord produced a significant increase in MEPs' amplitude with respect to motor imagery of a 10^th^ interval chord. (b) APB muscle showed greater facilitation than ECD and FDS. Similarly, ADM showed increased corticospinal excitability when compared to ECD. Bars denote standard errors. Asterisks indicate significant differences.

For the factor muscle, pairwise comparisons of MEP amplitude of ECD vs. APB (*p* < 0.001), ECD vs. ADM (*p* < 0.001), and FDS vs. APB (*p* < 0.001) reached significance. A trend toward significance was found for the comparison of FDS vs. ADM (*p* = 0.090) (Figure 2). Therefore, corticospinal excitability assessed during motor imagery of chords was greater at the APB compared to the ECD and FDS and at the ADM compared to the ECD. No significant differences in the corticospinal output emerged between the APB and the ADM nor between the ECD and the FDS.

The comparison of MEP amplitude recorded during motor imagery of the 9^th^ interval chord vs. the 10^th^ interval chord yielded a significant result (*p* = 0.01). Additionally, a trend toward significance was observed with respect to the comparison between the 6^th^ interval chord and the 9^th^ interval chord (*p* = 0.09). Overall, an increase of MEP amplitude was observed from the 5^th^ to the 9^th^ chord, although not significantly. Conversely, corticospinal excitability was significantly reduced during motor imagery of the 10^th^ chord compared to the 9^th^ chord (Figure 2).

### 3.2. Nonnegative Matrix Factorization and Synergy Extraction

**Figure 3:**
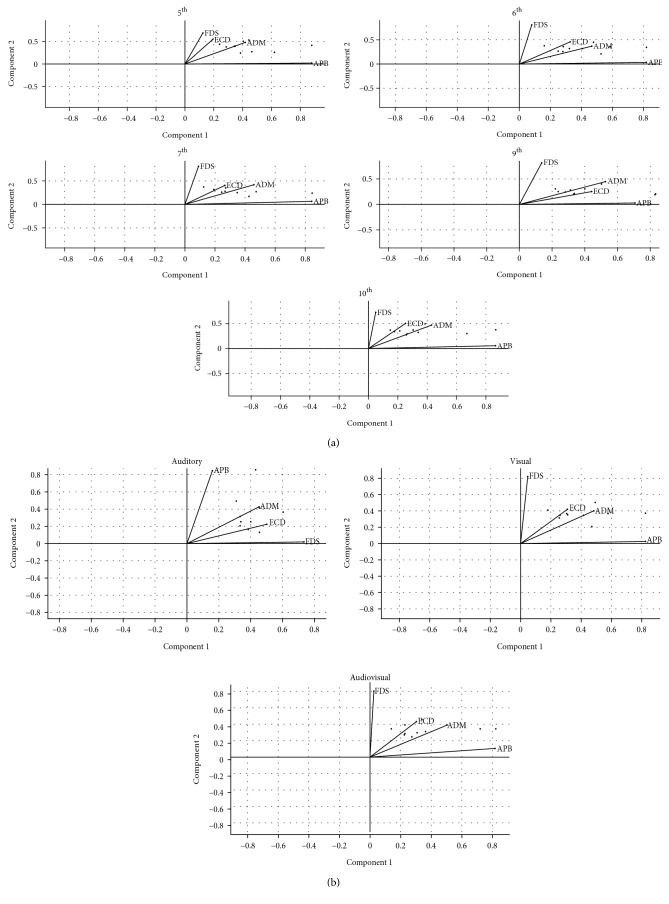
Synergies extracted by means of NNMF across different chords (a) and conditions (b). Line vectors indicate coefficients, whereas dots correspond to the values estimated in the synergy matrix (see text for further details).

As shown in Figure 3, following the extraction of 2 synergies, a VAF of 83.16%, 87.47%, 91.34%, and 94.17% in the 5^th^, 6^th^, 7^th^, 9^th^, and 10^th^ interval chords, respectively, emerged. The first component showed a greater coefficient for the APB than for the other muscles. Conversely, the ECD, FDS, and ADM were mainly represented in the second component. Pairwise comparisons of the synergies extracted across all chords never yielded significance using the 99^th^ percentile of the distribution of the shuffled dot products. However, all the dot products of the synergy's pairs in the first component and almost all in the second component fell within the 95^th^ percentile; the remaining ones distributed below the 90^th^ percentile.

**Figure 4:**
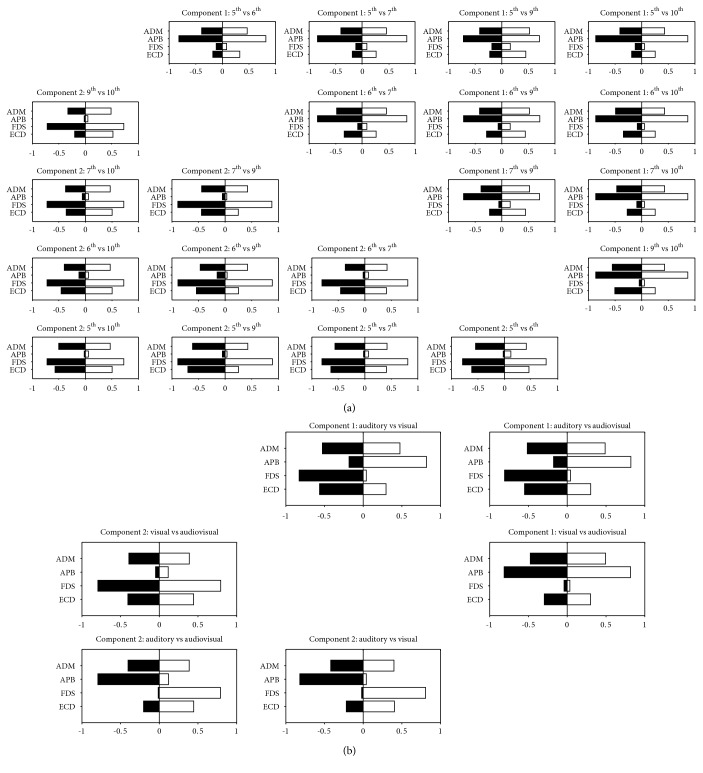
Pairwise comparisons of synergy coefficients calculated across different chords (a) and conditions (b).

VAF exceeded 90% after extraction of two synergies in all the three conditions (i.e., 92.61%, 90.80%, and 93.72% for the auditory, visual, and audiovisual condition, respectively). In the auditory condition, the APB was mainly represented in the first component, whereas ECD, FDS, and ADM in the first. The opposite situation was observed in the visual and audiovisual condition. Therefore, a significant difference emerged in pairwise comparisons of auditory vs. audiovisual and visual vs. audiovisual condition (Figure 4). Conversely, the dot products of coefficients distributed below the 95^th^ percentile of the shuffled dot product distribution with respect to the comparison of the visual vs. audiovisual condition. Despite the difference observed, it is worth to note that a similar segregation in different component was observed for the APB compare to ECD, FDS, and ADM.

## 4. Discussion

It is well known that some of the most worldwide famous piano players, as Horowitz, Schoenberg, and Rubinstein, successfully used motor imagery as musical training or immediately prior to a concert exhibition to improve proficiency in musical performance [35]. However, neuroimaging research has provided conflicting results on the functional involvement of the primary motor cortex in pianists performing musical imagery tasks [19, 21]. Taking into consideration that hand motor synergies (i.e., the patterns of muscle activity whose timing and amplitude modulation enable the correct production of movements [33, 36]) are encoded in the human primary motor cortex for hand gestures [7], such lack of motor cortex involvement during imagery is an unexpected finding that could depend upon several, not mutually exclusive, aspects: playing or imagining music require the allocation of most of functional resources for a dynamic integration of perceptual, cognitive, and emotional operations [37], so that activations of sensorimotor areas may remain hidden at a certain statistical mapping level. Notably, the motor executive aspects in expert musicians are obviously overlearned and somewhat automatized, so that they may require less functional activation of final common effector cortices [38, 39]: this would allow players to better concentrate on expressivity, emotions, or online control of the produced sounds. Obviously, not only the motor cortex controls motor synergies for hand gestures [7]: intracortical connections [40] as well as propriospinal branching of corticospinal axons are also regarded as the neural substrates of muscle synergies involved in coordinated multijoint movements [3, 40–42].

Nevertheless, converging neuroimaging and neurophysiological studies showed that the activity of the hand area of the motor cortex in piano players is increased during listening of familiar musical pieces [22, 43, 44] or during the observation of fingering errors on a keyboard [45]. However, there are no studies investigating by electrophysiological techniques the online modulation of corticospinal output for different muscle groups during motor imagery of chords in professional piano players and in musicians in general. This is precluding any step of knowledge regarding motor synergies used by musicians during musical planning/execution, as well as how these are modulated by audio and visual musical stimuli. This, despite the evidence that musical training promotes the emergence of audio- [43] or visuomotor [46–48] cross-modal activations in a frontoparietal network including the primary motor cortex [49]. Moreover, action sound and action observation of everyday hand gestures, congruent with the perceived action, have been already proven to produce selective corticospinal facilitation in normal subjects [50]. Hence, it is reasonable that professional piano players, in which a sort “musical grammar” for musical-related hand gestures is operating at cortical level [51, 52], might well capitalize from cross-modal perceptions to tune at the best of their motor output towards efficient motor synergy production, such as during very fine action representations required by progressively increasing triad chords intervals.

Current results show that professional pianists are able to cross-modally modulate their corticospinal output during the mental imagery of triad chords: motor imagery produced the highest corticospinal facilitation in the hand muscles rather than in the forearm ones. This is not surprising, as either the ABP or ADM was always engaged in the mental execution of each triad chord, although with different demands: the former remained stable on the C note and the latter was required to be “mentally extended.”

However, such fine tuning is sustained by a definite three-effector (at least) motor synergy including the FDS and ADM muscles (that represent the prime movers for the required progressive hand extension) as well as the ECD muscle that is coacting with the ADM muscle for the little finger abduction. A gradual increase, although not significant, in corticospinal excitability was observed moving from the 5^th^ interval chord to the 9^th^ interval chord across progressively wider imagined extensions of the hand. Since a trend toward significance was detected comparing the 6^th^ interval chord with the 9^th^ interval chord, the lack of significant differences between the other chords might reflect the fact that such intervals are the easiest to be recognized and “executed” for musicians, that the task did not engage sufficiently the primary motor cortex, or that the sample size was too small to make small changes statistically significant.

Not surprisingly, prompting chords using auditory, visual, or audiovisual musical stimuli produced similar effects on corticospinal excitability towards the prime mover muscles: this may indicate that professional musicians are able to translate and capitalize the musical information independently by the sensory channel (i.e., auditory or visual) used to acquire them (see [43, 48, 53]). Adding a cross-modal reinforcement (as in the audiovisual condition) did not result in additional modulation of the corticospinal system or motor synergy variations, suggesting that the corticospinal tuning was likely already working at its best in professional musicians even during monomodal presentation. However, since a control group of nonmusicians was not included in the study, this conclusion remains highly probable but speculative. Further studies are needed to disentangle the role of musical skillfulness in motor imagery.

Even if not investigated here, besides the primary motor cortex, the premotor cortex (PMC) might also be a candidate for the observed cross-modal tuning of corticospinal output in piano players. Indeed, electrophysiological studies in animals have shown that neurons in the PMC respond to auditory and visual stimuli that are linked to known actions [54] and, in humans, perturbation of PMC by repetitive TMS disrupts learning of listened melodies [55] or rhythmic entrainment [56]. PMC also plays a relevant role in visuomotor transformation in humans [57]. The PMC is closely connected with the primary motor cortex that according to recent neuroimaging evidence [7] encodes motor synergies for human hand gestures.

Language [43], music [58], and actions [59] share a common syntactic-like overlapping structure. Effects of audiovisual feedback on corticospinal output in piano players may be regarded as the analogue of the many physiologically demonstrable multisensory cross-modal interactions on the motor system: action observation [26, 27, 60–63] or action listening [64], speech listening [65–67], especially in the case of action-related words [68, 69], or smelling food [70], induce corticospinal facilitation in the muscles the actor would use to actually execute congruent actions. Moreover, it is known that neural representations of action-related sounds depend on motor familiarity [71], as chords for musicians. These facilitatory effects induced by audio- and visuomotor transformations could be particularly amplified in professional musicians [72], thanks to their enhanced functional [73] and structural [74] adaptive plastic capabilities in the sensorimotor brain areas.

It might be argued that an involuntary motor cortical activity may be elicited in piano players by music listening, especially for chords requiring the action of the thumb and of the little finger, and that these activities might have biased MEP amplitude. This effect is ruled out by the initial practice carried out by all subjects with the task and by continuous visual and acoustic monitoring of electromyographic activity in seconds preceding the TMS pulse throughout the experiment. Moreover, the musical-related specificity of the observed cross-modal effects on the engagement of specific motor synergies, but even on corticospinal output in general, rules out the possibility that corticospinal changes might be solely due to the peculiar pianists' skillfulness in finger motor abilities: it is unlikely, but it is a matter to be verified experimentally, that music-naïve typewriters or braille readers might undergo to similar music-related corticospinal effects, beyond the facilitation induced by motor practice alone.

## Data Availability

All data used to support the findings of this study are included within the article. Additional data can be requested to the corresponding author.
